# Association between duration of intravenous antibiotic administration and early-life microbiota development in late-preterm infants

**DOI:** 10.1007/s10096-018-3193-y

**Published:** 2018-01-24

**Authors:** Romy D Zwittink, Ingrid B Renes, Richard A van Lingen, Diny van Zoeren-Grobben, Prokopis Konstanti, Obbe F Norbruis, Rocio Martin, Liesbeth J M Groot Jebbink, Jan Knol, Clara Belzer

**Affiliations:** 1 Laboratory of Microbiology, Wageningen University, Stippeneng 4, 6708 WE Wageningen, The Netherlands; 2 Nutricia Research, Utrecht, The Netherlands; 3 Princess Amalia Children’s Centre, Department of Paediatrics and Neonatology, Isala, Zwolle, The Netherlands

## Abstract

Antibiotic treatment is common practice in the neonatal ward for the prevention and treatment of sepsis, which is one of the leading causes of mortality and morbidity in preterm infants. Although the effect of antibiotic treatment on microbiota development is well recognised, little attention has been paid to treatment duration. We studied the effect of short and long intravenous antibiotic administration on intestinal microbiota development in preterm infants. Faecal samples from 15 preterm infants (35 ± 1 weeks gestation and 2871 ± 260 g birth weight) exposed to no, short (≤ 3 days) or long (≥ 5 days) treatment with amoxicillin/ceftazidime were collected during the first six postnatal weeks. Microbiota composition was determined through 16S rRNA gene sequencing and by quantitative polymerase chain reaction (qPCR). Short and long antibiotic treat ment significantly lowered the abundance of *Bifidobacterium* right after treatment (*p* = 0.027) till postnatal week three (*p* = 0.028). Long treatment caused *Bifidobacterium* abundance to remain decreased till postnatal week six (*p* = 0.009). Antibiotic treatment was effective against members of the Enterobacteriaceae family, but allowed *Enterococcus* to thrive and remain dominant for up to two weeks after antibiotic treatment discontinuation. Community richness and diversity were not affected by antibiotic treatment, but were positively associated with postnatal age (*p* < 0.023) and with abundance of *Bifidobacterium* (*p* = 0.003). Intravenous antibiotic administration during the first postnatal week greatly affects the infant’s gastrointestinal microbiota. However, quick antibiotic treatment cessation allows for its recovery. Disturbances in microbiota development caused by short and, more extensively, by long antibiotic treatment could affect healthy development of the infant via interference with maturation of the immune system and gastrointestinal tract.

**Electronic supplementary material:**

The online version of this article (10.1007/s10096-018-3193-y) contains supplementary material, which is available to authorized users.

## Introduction

Over the last several years, the intestinal microbiota has been well recognised as a major contributor to human health and disease [3]. Development of the intestinal microbiota from birth to childhood and adulthood is a process that co-occurs with maturation of the immune, digestive and cognitive systems. The interplay between humans and their intestinal microbes could, therefore, greatly influence health, especially during critical developmental stages in early life. Despite its described importance, intestinal microbiota development is not completely understood, as it is a highly dynamic process affected by multiple host and environmental factors, of which gestational age, mode of delivery, diet and antibiotics are perceived as the major influencing factors [29]. Previous studies showed that antibiotic treatment in early life can lead to short- and long-term alterations of the intestinal microbiota, which has been related to early and later life health outcomes such as asthma and adiposity [8, 19, 32].

Antibiotic treatment is common practice in the neonatal ward for the prevention and treatment of sepsis, which is one of the leading causes of mortality and morbidity in preterm infants. Antibiotics, such as amoxicillin, ceftazidime, erythromycin and vancomycin, are frequently used as they target a broad spectrum of pathogens. Intrauterine infections are a common cause of preterm birth; thus, many preterm infants are born with suspicion of infection and are, therefore, treated with antibiotics from birth onwards. In preterm infants, group B streptococci and *Escherichia coli* are associated with onset of neonatal sepsis [31]. Since sepsis in preterm infants still has a high mortality and morbidity, it is not possible to wait for test results before starting antibiotic treatment. To reduce the antibiotic load in the neonatal ward, it is common practice to evaluate the need for antibiotics after 48 h and stop antibiotics if the infection is not proven.

The applied antibiotic strategies in neonatology led to decreased mortality and morbidity rates. However, there is a risk of impeding gut microbiota development and increasing antibiotic resistance [15]. It has been shown that, in infants, intestinal microbiota composition and activity in early life is associated with gestational age and can be related to the degree of perinatal antibiotic administration [1, 34]. In addition, it has been shown that preterm infants admitted to the neonatal intensive care unit (NICU) are particularly colonised by antibiotic-resistant and virulent bacterial strains during early life, which was restored around two years of age [24]. Despite increased understanding of how antibiotic treatment affects preterm infant microbiota development, not much is known about the effect of duration of treatment. Previous studies showed that long antibiotic treatment (> 5 days) in preterm infants results in lower diversity of the faecal microbiota than short treatment, but no clear differences in overall microbiota composition were observed [9, 17]. This could be due to other factors that influence microbiota composition which were not accounted for during stratification of the infants, such as gestational age [17] or mode of delivery [9]. As gestational age and mode of delivery by themselves influence early-life microbiota development, the present study accounted for these in order to further understand the effect of antibiotic treatment duration on preterm infant microbiota development.

## Materials and methods

### Subjects and sample collection

This study was part of an observational, non-intervention study involving (pre)term infants admitted to the hospital level III NICU or the level II neonatal ward of Isala in Zwolle, the Netherlands. The ethics board from METC Isala Zwolle concluded that this study does not fall under the scope of the Medical Research Involving Human Subjects Act (WMO). Informed consent was obtained from both parents of all individual participants included in the study. For faecal microbiota profiling, 15 late preterm infants (mean ± standard deviation [SD], 35.7 ± 0.9 weeks gestation, 2871 ± 261 g birth weight) were longitudinally sampled during the first six postnatal weeks, resulting in a total of 95 samples. Sampling days for each infant can be found in Table 1. Infants received either no (control), short-term (ST, < 3 days) or long-term (LT, > 5 days) treatment with a combination of amoxicillin and ceftazidime during the first postnatal week. Infants started antibiotic treatment on clinical suspicion of a bacterial infection and, upon negative or positive cultures. Antibiotic administration was respectively stopped (ST) after two to three days or continued (LT) up till a maximum of seven days. Of the LT infants, one was diagnosed with sepsis and three with pneumonia, and, in all cases, the causative pathogen was unknown. Meconium and faecal samples were collected at birth and at postnatal weeks one, two, three, four and six. Samples were stored temporally at − 20 °C until transfer to − 80 °C. Infant clinical characteristics can be found in Table 1. All infants were born vaginally, only received enteral nutrition and did not have clinical signs of food intolerance.

**Table 1:** Infant clinical characteristics

Group	Infant	Gender	GA	BW (g)	AB duration (days)	Reason AB	Maternal AB	Days until FEF	Human milk (% per week)	Sampling days	Discharge	PREE	PROM	Drip	Pain medication	Antimycotics
Control	A	Female	35 + 2	2700	0		No	8	75, 98, 27, 0, 0	2, 7, 16, 22, 29, 39	9	No	Yes	No	No	No
Control	B	Male	35 + 5	3110	0		No	7	70, 100, 100, 100, 100	3, 5, 14, 21, 28, 42	5	No	No	No	No	No
Control	C	Male	34 + 2	2800	0		Yes	9	90, 100, 100, 100, 100	1, 6, 13, 21, 29, 41	12	No	No	Yes	No	Yes
Control	D	Male	35 + 1	3030	0		No	7	62, 100, 100, 100, 100	1, 6, 14, 22, 29, 43	11	No	No	No	Yes	No
Control	E	Male	35 + 1	2500	0		No	6	90, 100, 100, 100, 100	4, 6, 14, 21, 28, 43	8.8 ± 2.6	Yes	No	No	Yes	No
Control average ± SD			36.4 ± 0.5	2828 ± 221				7.4 ± 1.0			8.8 ± 2.6					
ST	F	Male	34 + 5	2385	3	Suspicion	Yes	7	30, 0, 0, 0, 0	3, 6, 7, 14, 22, 29, 44	10	No	Yes	Yes	No	No
ST	G	Male	35 + 2	3050	2.5	Suspicion	No	6	63, 100, 100, 100, 100	3, 6, 14, 21, 28, 42	6	No	No	Yes	No	No
ST	H	Male	35 + 2	2515	3	Suspicion	Yes	7	93, 100, 100, 100, 100	2, 6, 14, 21, 29, 43	10	No	No	Yes	No	Yes
ST	I	Male	37 + 0	2980	2	Suspicion	Yes	6	69, 89, 90, 86, 88	1, 4, 7, 14, 21, 28, 43	5	No	Yes	Yes	No	No
ST	J	Female	37 + 1	3130	2	Suspicion	No	7	80, 100, 100, 100, 100	2, 6, 13, 20, 29, 42	4	Yes	No	Yes	Yes	No
ST average ± SD			35.8 ± 1.0	2812 ± 302				6.6 ± 0.5			7.0 ± 2.5					
LT	K	Male	36 + 6	2930	7	Pneumonia	No	6	74, 100, 100, 100, 47	3, 6, 10, 14, 21, 28, 42	8	No	Yes	Yes	No	Yes
LT	L	Female	35 + 3	2903	6	Sepsis	No	7	94, 100, 100, 100, 100	3, 6, 10 15, 21, 28, 42	15	No	No	Yes	No	Yes
LT	M	Male	34 + 5	2805	5	Suspicion sepsis	Yes	6	22, 20, 19, 29, 29	4, 8, 14, 22, 28, 43	6	No	Yes	Yes	Yes	No
LT	N	Male	36 + 1	2830	7	Pneumonia	No	8	67, 97, 100, 100, 100	1, 5, 12, 20, 27, 41	14	No	Yes	Yes	Yes	No
LT	O	Male	37 + 1	3400	7	Pneumonia	No	7	7, 33, 25, 11, 10	4, 6, 11, 14, 21, 28, 42	8	Yes	No	Yes	No	No
LT average ± SD			36.1 ± 0.9	2974 ± 218				6.8 ± 0.7			10.2 ± 3.6					

GA: gestational age; BW: birth weight; AB: antibiotics; FEF: full enteral feeding; PREE: pre-eclampsia; PROM: prolonged rupture of membranes

### DNA extraction

DNA was extracted from faeces by the repeated bead beating plus phenol/chloroform method, as described previously [23]. DNA was quantified using a NanoDrop ND-2000 spectrophotometer (Thermo Fisher Scientific, Wilmington, DE, USA) and by using a Qubit® 2.0 Fluorometer (Life Technologies, Carlsbad, CA, USA), according to manufacturers’ instructions.

### 454 pyrosequencing

Amplification of the V3–V5 regions of the 16S rRNA gene was performed using the *Bifidobacterium*-optimised 357F and 926Rb primers, as described previously [30]. For each sample, the reverse primer included a unique barcode sequence to allow for multiplexing. Polymerase chain reaction (PCR) and 454 pyrosequencing (GS Junior, Roche) were performed by LifeSequencing S.L. (Valencia, Spain), as described previously [30]. Sequencing data are available in the European Nucleotide Archive (http://www.ebi.ac.uk/ena) under study accession PRJEB19937.

### Sequencing data analysis

Pyrosequencing data were analysed using the QIIME software package (v1.8) [6] applying Acacia [5], USEARCH [12], UCLUST [11] and the SILVA 111 database [28] for denoising, chimera removal, operational taxonomic unit (OTU) picking and taxonomic classification, respectively. The obtained OTU table was filtered for OTUs with a number of sequences less than 0.005% of the total number of sequences [4]. To account for variation between samples’ total number of reads, rarefaction to 4085 reads per sample was applied.

To identify bacterial taxa that were significantly different in abundance between control, ST and LT infants, the non-parametric Kruskal–Wallis test with Monte Carlo permutation (10,000×) was applied. The Kruskal–Wallis test was done using absolute read counts for each taxonomic group and after the OTU table was filtered for OTUs present in less than 25% of the samples. To compare richness and diversity between samples, the Wilcoxon signed-rank test, the Mann–Whitney *U*-test and Kruskal–Wallis test were applied for dependent, two groups of independent and more than two groups of independent samples, respectively. To study (dis)similarities in microbiota composition and relate changes in microbiota composition to clinical data, principal component analysis (PCA) and redundancy analysis (RDA) were performed using the Canoco multivariate statistics software v5. For RDA, factors were considered significant when the Bonferroni-corrected *p*-value was below 0.05. Co-occurrence patterns were determined by Spearman correlation using the taxa that remained after the OTU table was filtered for OTUs present in less than 25% of the samples. Visualisation was done using the Gephi-0.9.1 platform (https://gephi.org) and Adobe Illustrator CS6.

### qPCR analysis

Real-time PCR amplification and detection were performed on a CFX384™ real-time PCR detection system (Bio-Rad). The reaction mixture was composed of 5 μL iQ™ SYBR® Green Supermix, 0.2 μL forward and reverse primers (10 nmol), 1.6 μL nuclease-free water and 3 μL of DNA template (2 ng/μL). Primers used targeted total 16S [25], *Bifidobacterium* [10], *Enterococcus* [22] and Enterobacteriaceae [26]. The program for amplification of total 16S, *Bifidobacterium* and *Enterococcus* was initial denaturation at 94 °C for 5 min, followed by 40 cycles of denaturation at 94 °C for 20 s, annealing at 60 °C for 20 s and elongation at 72 °C for 50 s, followed by a melt-curve from 60 °C to 95 °C with 0.5 °C steps. The program for amplification of Enterobacteriaceae was initial denaturation at 95 °C for 5 min, followed by 40 cycles of denaturation at 95 °C for 10 s, annealing at 55.8 °C for 20 s and elongation at 72 °C for 20 s, followed by a melt-curve from 60 °C to 95 °C with 0.5 °C steps. Standard curves contained 10^1^–10^9^ 16S rRNA copies/μL and were performed in triplicate.

Data were analysed using the CFX Manager™ software (Bio-Rad). Relative abundances of the taxa were determined by dividing the taxa-specific 16S rRNA gene copy number by the total 16S rRNA gene copy number. Quantitative polymerase chain reaction (qPCR) and pyrosequencing data had a Spearman correlation of 0.758, 0.729 and 0.822 for *Bifidobacterium*, *Enterococcus* and Enterobacteriaceae, respectively. To identify bacterial taxa that were significantly different in abundance between control, ST and LT infants, the non-parametric Kruskal–Wallis test with Monte Carlo permutation (10,000×) was applied.

## Results

### Antibiotic treatment delays colonisation by *Bifidobacterium*

**Fig. 1:**
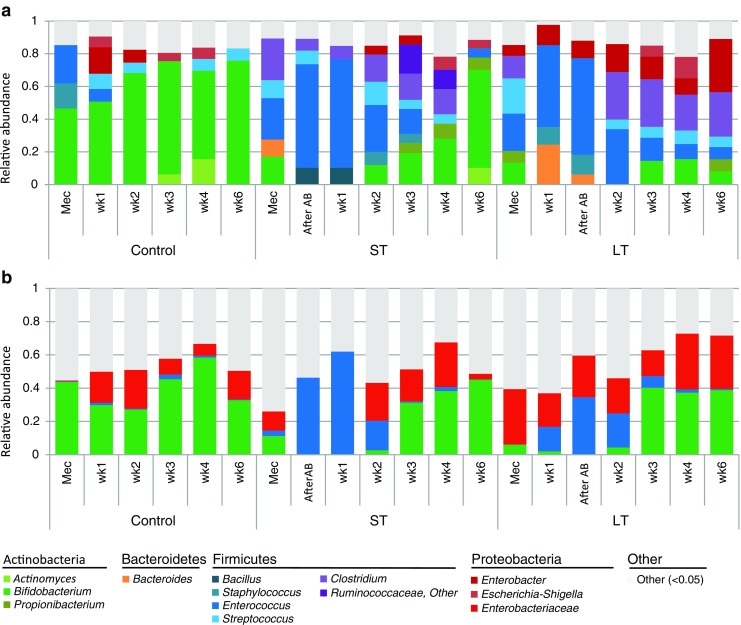
Microbiota composition profiles based on 16S rRNA gene sequencing (**a**) and quantitative polymerase chain reaction (qPCR) (**b**). Per time point, the averages of five infants are shown. For 16S rRNA gene sequencing data, genera with a relative abundance of more than 5% are shown

Microbiota composition throughout the first six postnatal weeks was determined in 15 infants with varying antibiotic treatment duration. The microbiota composition of control infants was characterised by a high abundance of *Bifidobacterium* throughout the first six postnatal weeks, with an average relative abundance of 45% in meconium, increasing towards 73% at postnatal week six (Fig. 1a). In three out of five infants, *Bifidobacterium* already covered more than 50% abundance in the meconium sample (Online Resource 1). These findings were confirmed by qPCR analysis (Fig. 1b, Online Resource 2). Despite common characteristics, control infants’ microbiota composition also contained individual specific profiles. Outstanding were: dominance of *Enterobacter* at postnatal weeks one and two in one infant (infant C), dominance of *Actinomyces* at postnatal weeks three and four in another infant (infant B), members of Proteobacteria were only identified in three infants and, in three infants, *Enterococcus* was a dominant member in the meconium sample (Online Resource 1). The microbiota composition of ST and LT infants was not characterised by a particular microbiota profile that lasted throughout the first six postnatal weeks, but showed high variability between and within infants (Fig. 1a, Online Resource 1).

To understand the effect of antibiotic treatment duration on intestinal microbiota development, the microbiota composition was compared between control, ST and LT infants. ST and LT infants had a significantly lower abundance of *Bifidobacterium* right after antibiotic treatment (*p* = 0.027, 0.027) and at postnatal weeks one (*p* = 0.027, 0.021), two (*p* = 0.016, 0.009) and three (*p* = 0.028, 0.028) compared to control infants. In LT infants, *Bifidobacterium* abundance also remained lower during postnatal weeks four (*p* = 0.086) and six (*p* = 0.009), whereas this could not be observed in ST infants. qPCR analysis confirmed the significantly lower quantity of *Bifidobacterium* right after a short (*p* = 0.033) or long (*p* = 0.035) antibiotic treatment compared to control infants. *Enterococcus* became a dominant member of the community in multiple ST and LT infants during the first postnatal week, which was not observed in any of the control infants (Fig. 1, Online Resources 1 and 2); however, except for postnatal week two, differences were not statistically significant. The total bacterial count, as determined by qPCR, was not significantly reduced by antibiotic treatment (Online Resource 3). However, in some ST and LT infants, lower total bacterial count at early time points suggests delayed colonisation due to antibiotic treatment.

**Fig. 2:**
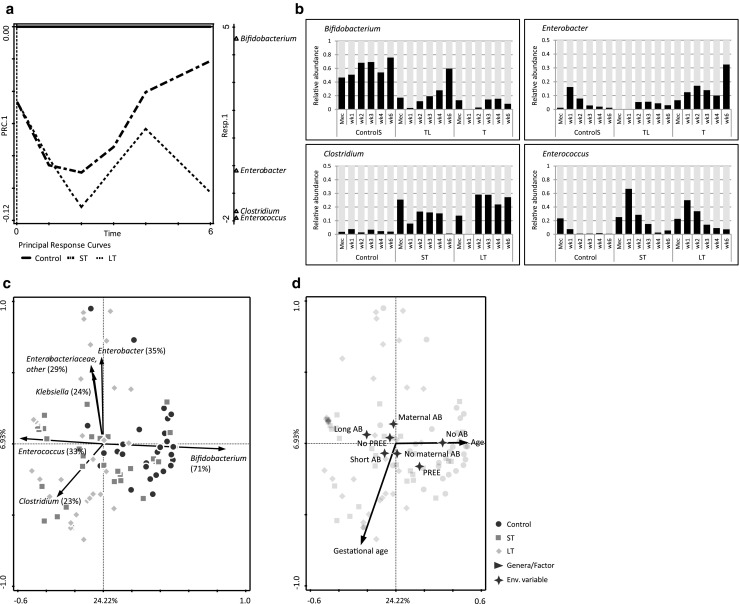
Principal response curve (PRC) and redundancy analysis (RDA) of the faecal microbiota in control, short-term (ST) and long-term (LT) infants. **a** PRC analysis. Genera with a score lower than − 0.5 or higher than 0.5 are shown on PRC1 (*Bifidobacterium*: 4.58; *Enterococcus*: − 1.83; *Clostridium*: − 1.70; *Enterobacter*: − 0.73). **b** Relative abundance of the bacterial genera associated with temporal development as observed in the PRC. Per time point, the average relative abundance is shown. **c** RDA showing the main bacterial genera explaining the variation. Percentages indicate the fit of the bacterial genera into the ordination space. Genera with a fit over 20% are shown. **d** RDA showing the clinical factors associated with microbiota composition. Clinical factors that significantly (*p* < 0.05) explain the variation are shown. AB: antibiotics; PREE: pre-eclampsia

The differences in microbiota development over time were further explored via principal response curve (PRC) analysis and RDA. Temporal microbiota development was different between control, ST and LT infants (*p* = 0.002) (Fig. 2a). Short treatment allowed for development towards a microbiota composition more similar to control infants, characterised by a high abundance of *Bifidobacterium* (Fig. 2a, c). Abundances of *Bifidobacterium*, *Clostridium* and *Enterococcus* were different between control and antibiotic-treated infants (Fig. 2b). The abundance of *Bifidobacterium* and *Enterobacter* at postnatal week six explained the difference in temporal microbiota development between ST and LT infants (Fig. 2b). Antibiotic treatment duration was the main factor significantly explaining the observed variation in microbiota composition between samples (*p* = 0.002, Fig. 2d). No, short and long antibiotic treatment explained 14.9%, 3.6% and 3.6% of the variation, respectively. Other factors significantly explaining the variation were postnatal age (7.5%), gestational age (4.9%), pre-eclampsia (3.2%) and maternal antibiotics (2.9%) (Fig. 2d). Gender, birth weight, proportion of human milk, days until full enteral feeding, days until discharge, indwelling catheters, pain medication, antimycotic use and prolonged rupture of membranes did not significantly affect microbiota composition.

### Microbiota community structure is associated with its dominating taxa

**Fig. 3:**
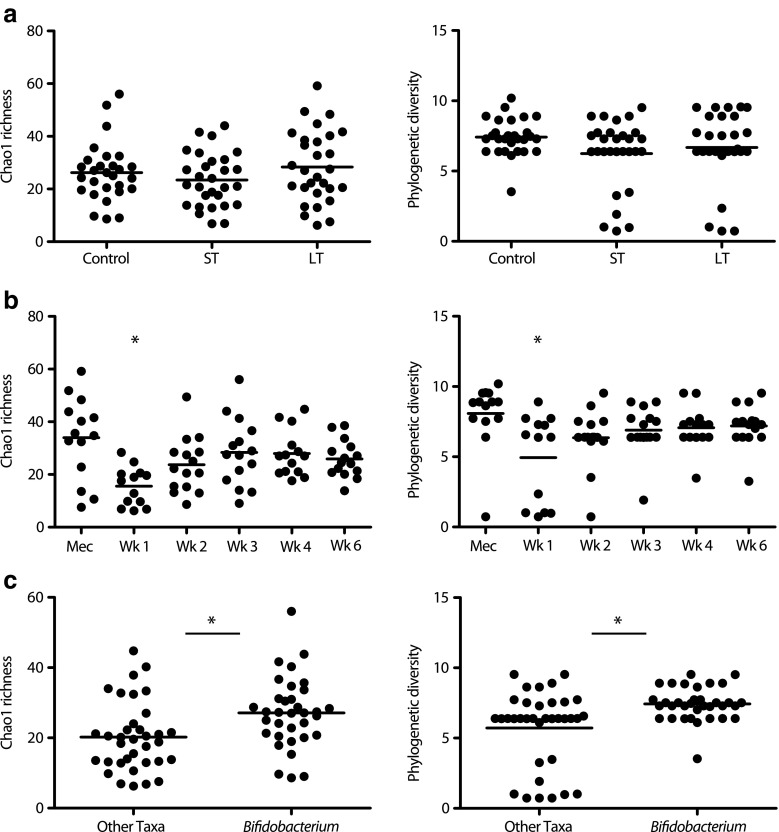
Bacterial community richness and diversity. **a** Samples stratified on antibiotic treatment duration. No significant difference observed. **b** Samples stratified on sampling time point. Samples at postnatal week one were significantly lower compared to all other time points (*p < 0.05; Mann–Whitney *U*-test with Monte Carlo permutation). **c** Samples stratified on dominating taxa (*p < 0.01; Mann–Whitney *U*-test with Monte Carlo permutation)

**Fig. 4:**
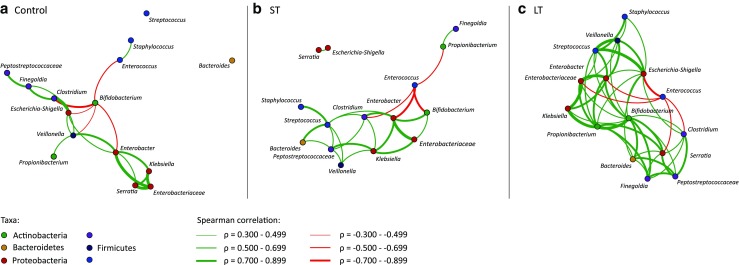
Co-occurrence patterns of the bacterial community in control (**a**), ST (**b**) and LT (**c**) infants. Patterns are based on significant (*p* < 0.05) Spearman correlations between genera

Short and long antibiotic treatment did not affect community richness and diversity (Fig. 3a, Online Resource 4). Instead, community richness and diversity were related to postnatal age, and depended on which taxa was dominant in the community (Fig. 3b, c). It was observed that one of the major differences in microbiota composition between control, ST and LT infants was expressed by the dominance of either *Bifidobacterium* or another genus like *Enterococcus*, *Enterobacter* or *Clostridium*. The dominance of *Bifidobacterium* in the bacterial community was related to significantly increased richness and diversity (Fig. 3c). To increase the understanding of bacterial community structure dynamics in control, ST and LT infants, co-occurrence patterns based on Spearman correlation were visualised (Fig. 4). In control infants, abundance of *Bifidobacterium* was negatively correlated to *Enterococcus*, *Veillonella*, *Clostridium*, *Escherichia*–*Shigella* and *Enterobacter*. In ST infants, *Enterococcus* was negatively correlated to *Bifidobacterium*, *Propionibacterium*, *Clostridium* and *Enterobacter*. In LT infants, *Enterococcus* was negatively correlated to *Clostridium*, *Serratia*, *Escherichia*–*Shigella*, *Enterobacter* and other Enterobacteriaceae.

## Discussion

Intravenous antibiotic administration for the prevention and treatment of infection and sepsis occurs frequently in preterm infants during the neonatal period. Therefore, studying the side effects of antibiotic treatment, including the effect on microbiota development, is of great relevance. The idea that short antibiotic use negatively affects clinical success and induces antibiotic resistance is gradually being replaced by the aim to avoid antibiotic overuse [20]. In this study, we focused on the effect of intravenous antibiotic treatment duration on intestinal microbiota development in preterm infants during the first six postnatal weeks. Our main findings are: (1) both short and long treatment with amoxicillin/ceftazidime during the first postnatal week drastically disturbed the normal colonisation pattern; (2) short, but not long, antibiotic treatment allowed for the recovery of *Bifidobacterium* levels within the first six postnatal weeks; and (3) community richness and diversity were not affected by antibiotic treatment, but were associated with postnatal age and with the dominance of specific bacterial taxa, leading to differences in microbial networks.

In the current study, 16S rRNA gene sequencing and qPCR analysis showed that preterm infants’ faecal microbiota was dominated by *Bifidobacterium* throughout the first six postnatal weeks. *Bifidobacterium* species are considered beneficial early-life colonisers, and are found in high abundance in term, vaginally delivered, breast-fed infants [27]. Short and long treatment with a combination of amoxicillin and ceftazidime during the first postnatal week drastically disturbed the normal colonisation pattern. Antibiotic treatment was effective against members of the Enterobacteriaceae family, but also negatively affected *Bifidobacterium* abundance and allowed *Enterococcus* to thrive. It must be noted that *Bifidobacterium* abundance was already lower in meconium samples of ST and LT infants compared to control infants, most likely a result of the relatively late (postnatal day 2–4) defecation of meconium samples by most preterm infants. *Enterococcus* remained dominant for up to two weeks after antibiotic treatment discontinuation. This might possess a health risk for the infants, as some *Enterococcus* species emerged from gut commensals to nosocomial pathogens via the acquisition of multi-drug resistance and other virulence determinants [2, 16]. Short, but not long, antibiotic treatment allowed for the recovery of *Bifidobacterium* levels within the first six postnatal weeks. Although the differences in average *Bifidobacterium* abundance between ST and LT infants was less apparent using qPCR instead of sequencing, it did show that *Bifidobacterium* levels recovered in 4/5 ST and in only 2/5 LT infants. In addition, both methods indicate that long antibiotic treatment results in increased abundance of members of the Enterobacteriaceae family at postnatal week six.

Antibiotic treatment did not affect community richness and diversity. However, richness and diversity were affected by postnatal age and by the dominance of specific bacterial taxa. Dominance of *Bifidobacterium* was negatively associated with abundance of other bacterial genera. Its dominance, however, allowed for higher community richness and diversity compared to dominance by other bacterial genera such as *Enterococcus*. We speculate that *Bifidobacterium* species control, but not outcompete, other bacterial species and that the microbial networks associated with *Bifidobacterium* species can, therefore, play an important role in early-life tolerance induction and immune system maturation. Microbiota profiles associated with antibiotic treatment could negatively influence immune system maturation via disturbance of the normal colonisation pattern. Indeed, previous studies showed that early-life antibiotic exposure increased susceptibility to immune-related diseases such as asthma and allergy, and associated this with perturbations in microbial composition [14, 33].

In addition to antibiotic treatment, our findings show that postnatal age, gestational age, pre-eclampsia and maternal antibiotics influenced microbiota composition. The latter two highlight the importance of maternal health status on infant microbiota development. Previous studies showed that microbes can be vertically transmitted, and that maternal health status, such as bodyweight and antibiotic use, affect infant microbiota development [7, 13, 21]. Maternal antibiotics could affect infant microbiota composition via prenatal exposure of the foetus to antibiotics, via alteration of the mother’s microbiota and, therefore, the inoculum at birth, and via transfer of antibiotics through breastfeeding. In the study described herein, the use of perinatal antibiotics was unevenly distributed among the study groups. This, in addition to the relatively small sample size, hindered to unravel the true impact of maternal antibiotics on infant microbiota development. Pre-eclampsia is a condition characterised by high blood pressure and proteinuria, and is associated with maternal and neonatal morbidity and mortality, preterm birth and intrauterine growth restriction [18]. The aetiology of pre-eclampsia is unknown, but this disorder could be linked to genetic factors, obesity, abnormal formation of placental blood vessels and autoimmune disorders [18]. Our findings suggest that pre-eclampsia or its accompanying conditions are associated with infant microbiota composition. However, the relation between pre-eclampsia and infant microbiota development needs to be further elucidated, as this study was not designed for studying this matter.

Overall, our findings show that intravenous antibiotic administration during the first postnatal week greatly affects the gastrointestinal microbiota community structure in preterm infants. However, quick cessation of antibiotic treatment allows for recovery of the microbiota. Disturbances in microbiota development caused by short and, more extensively, by long antibiotic treatment could affect healthy development of the infant via interference with maturation of the immune system and gastrointestinal tract. Clinicians should be aware of the disturbances that antibiotic treatment can cause and be strict in discontinuing antibiotic treatment as soon as possible to allow for a fast recovery of the microbiota community structure.

## Electronic supplementary material

Below is the link to the electronic supplementary material.

ESM 1 (DOCX 880 kb)
