# Supplementary material for: Association between duration of intravenous antibiotic administration and early-life microbiota development in late-preterm infants
Source: Eur J Clin Microbiol Infect Dis. 2018 Jan 24;37(3):475–83. doi: 10.1007/s10096-018-3193-y (PMC5816780; doi:10.1007/s10096-018-3193-y)
Supplement: Supplementary file 1 — (DOCX 880 kb) [file 10096_2018_3193_MOESM1_ESM.docx]

**SUPPLEMENTary Material**

**Association between duration of intravenous antibiotic administration and early life microbiota development in late-preterm infants.**

Romy D Zwittink^1^; Ingrid B Renes^2^; Richard A van Lingen^3^; Diny van Zoeren-Grobben^3^; Prokopis Konstanti^1^; Obbe Norbruis^3^, Rocio Martin^2^; Liesbeth J Groot Jebbink^3^; Jan Knol^1,2,X^; Clara Belzer^1,X^

**Content:**

**Online Resource 1**: Microbiota composition profiles based on 16S-rRNA gene sequencing in control (A), ST (B) and LT (C) infants. Genera with a relative abundance of more than 5% are shown.

**Online Resource 2**: Microbiota composition profiles based on real-time qPCR data in control (A), ST (B) and LT (C) infants.

**Online Resource 3**: Total bacterial count as determined by real-time qPCR. No significant difference observed between gestational age groups at each time point as determined by the Kruskal-Wallis test with Monte Carlo Permutation.

**Online Resource 4**: Bacterial richness and diversity in control, ST and LT infants during the first six postnatal weeks. No significant difference observed between gestational age groups at each time point as determined by the Kruskal-Wallis test with Monte Carlo Permutation.

**Online Resource 1. Microbiota composition profiles based on 16S-rRNA gene sequencing in control (A), ST (B) and LT (C) infants.** Genera with a relative abundance of more than 5% are shown.


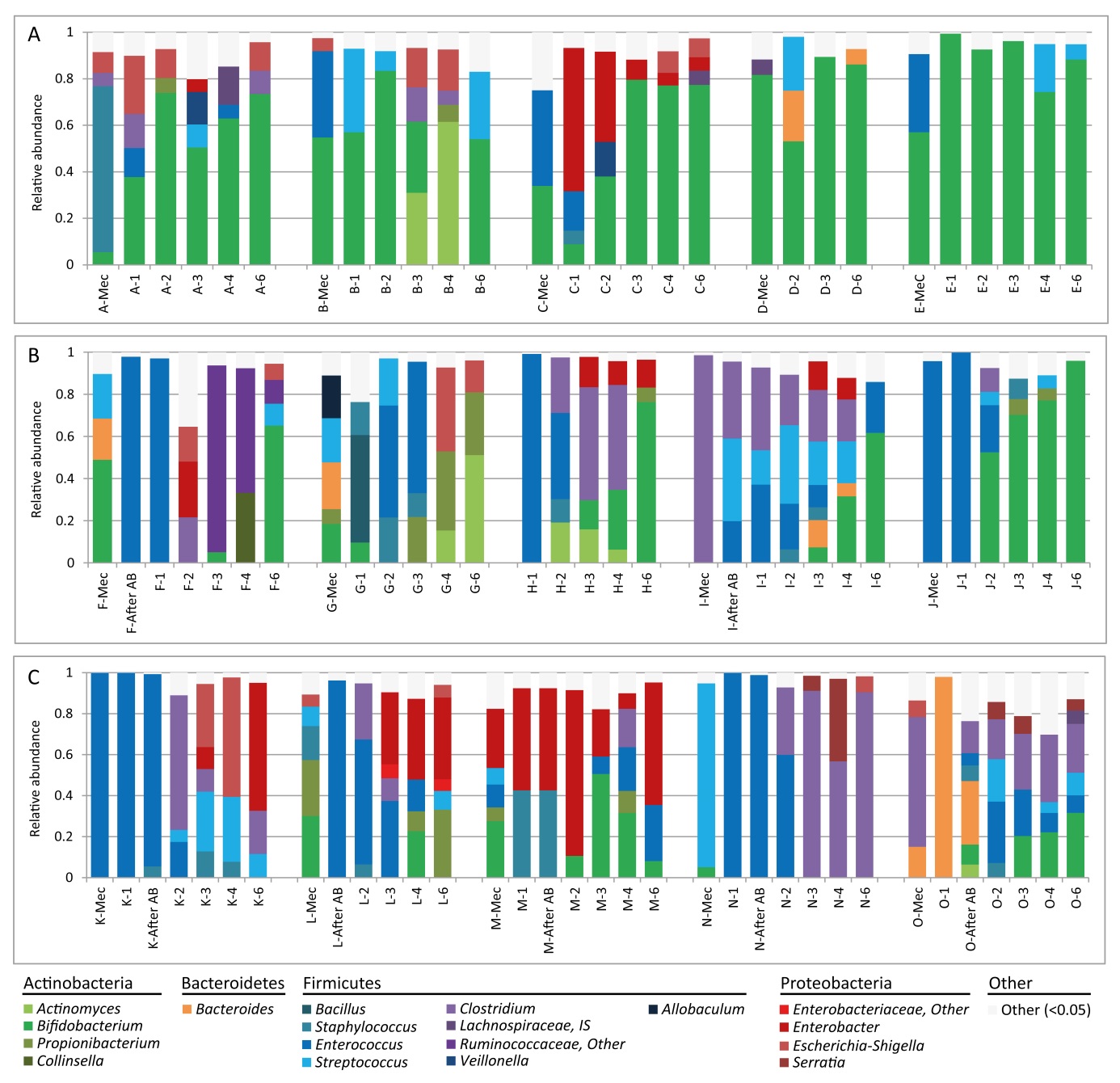


**Online Resource 2. Microbiota composition profiles based on real-time qPCR data in control (A), ST (B) and LT (C) infants.**


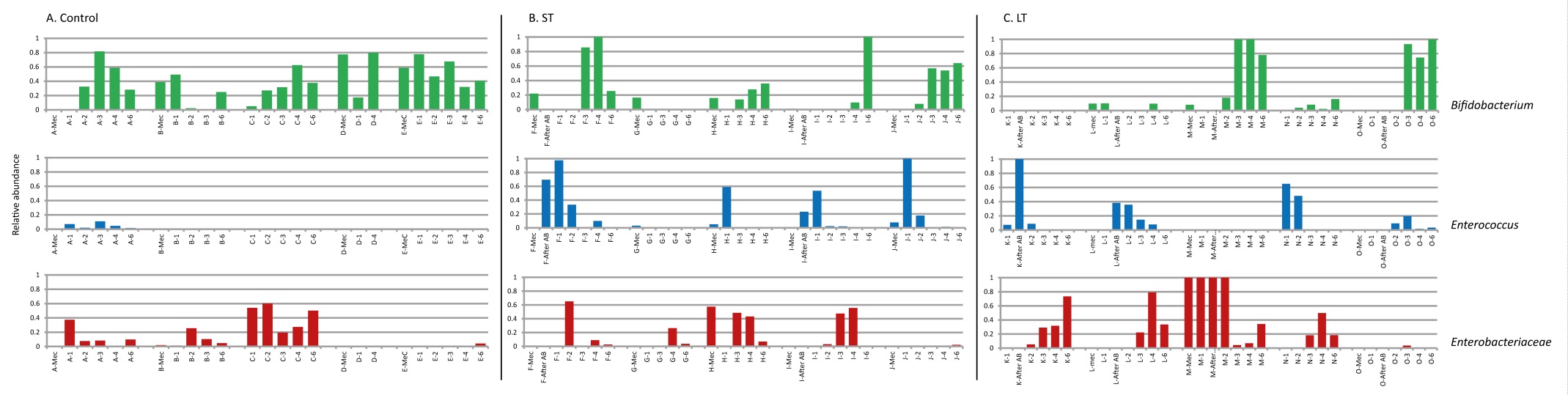


**Online Resource 3: Total bacterial count as determined by real-time qPCR.** No significant difference observed between gestational age groups at each time point as determined by the Kruskal-Wallis test with Monte Carlo Permutation.

**
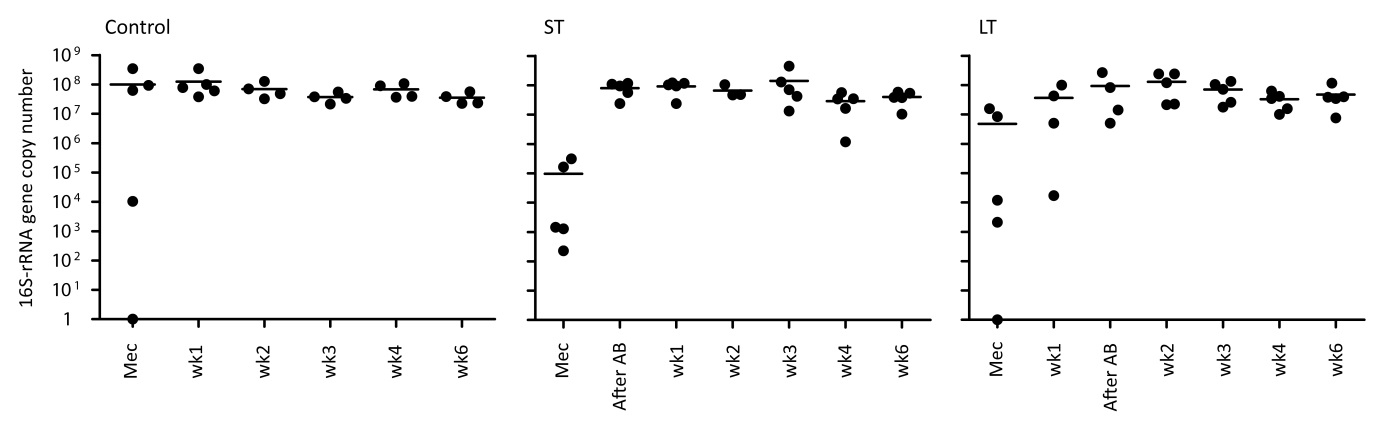
**

**Online Resource 4. Bacterial richness and diversity in control, ST and LT infants during the first six postnatal weeks.** No significant difference observed between gestational age groups at each time point as determined by the Kruskal-Wallis test with Monte Carlo Permutation.

**
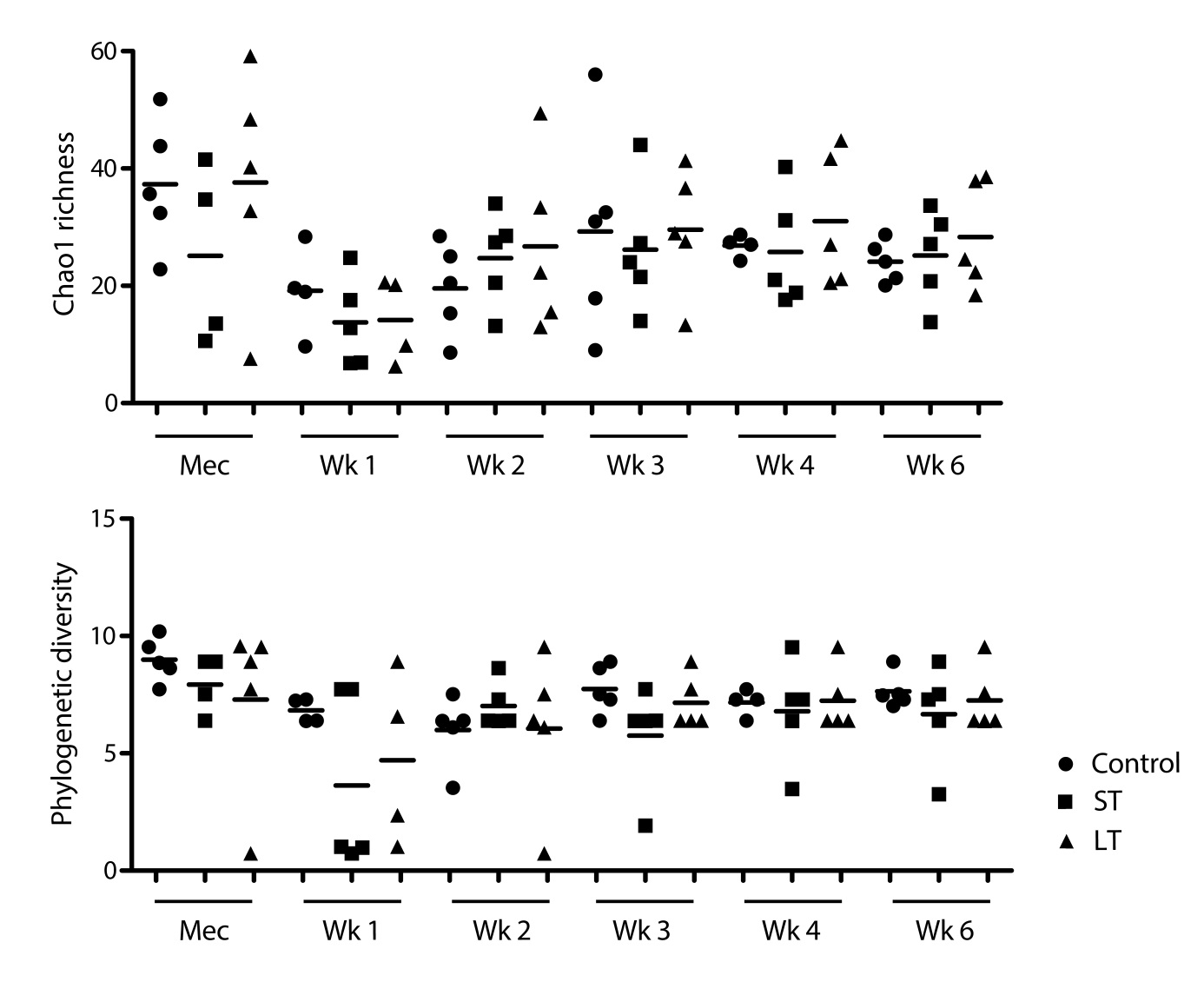
**
